# Super Sub-Nyquist Single-Pixel Imaging by Means of Cake-Cutting Hadamard Basis Sort

**DOI:** 10.3390/s19194122

**Published:** 2019-09-23

**Authors:** Wen-Kai Yu

**Affiliations:** 1 Center for Quantum Technology Research, School of Physics, Beijing Institute of Technology, Beijing 100081, China; yuwenkai@bit.edu.cn; 2 Key Laboratory of Advanced Optoelectronic Quantum Architecture and Measurements of Ministry of Education, School of Physics, Beijing Institute of Technology, Beijing 100081, China

**Keywords:** single-pixel imaging, compressed sensing, ghost imaging, Hadamard matrix, sub-Nyquist sampling, image reconstruction

## Abstract

Single-pixel imaging via compressed sensing can reconstruct high-quality images from a few linear random measurements of an object known a priori to be sparse or compressive, by using a point/bucket detector without spatial resolution. Nevertheless, random measurements still have blindness, limiting the sampling ratios and leading to a harsh trade-off between the acquisition time and the spatial resolution. Here, we present a new compressive imaging approach by using a strategy we call cake-cutting, which can optimally reorder the deterministic Hadamard basis. The proposed method is capable of recovering images of large pixel-size with dramatically reduced sampling ratios, realizing super sub-Nyquist sampling and significantly decreasing the acquisition time. Furthermore, such kind of sorting strategy can be easily combined with the structured characteristic of the Hadamard matrix to accelerate the computational process and to simultaneously reduce the memory consumption of the matrix storage. With the help of differential modulation/measurement technology, we demonstrate this method with a single-photon single-pixel camera under the ulta-weak light condition and retrieve clear images through partially obscuring scenes. Thus, this method complements the present single-pixel imaging approaches and can be applied to many fields.

## 1. Introduction

Techniques for capturing two-dimensional (2D) image information are significant for many applications, such as astronomical observation [1], phase retrieval [2], and fluorescence microscopy [3]. Single-pixel imaging (SPI), as one of these imaging techniques, offers many benefits especially for the scenarios where pixelated array detectors are too expensive and not well developed, having drawn more and more attention. To our knowledge, SPI might trace back to early raster scanning modalities, such as the flying-spot camera in 1884 and optical coherence tomography [4] in 1991. The existing mature array detectors mostly apply this point scanning technology. As specified by the Nyquist–Shannon criterion, the number of discrete measurements of an image of p×q=N pixels should be *N*, i.e., it requires full sampling. Subsequently, ghost imaging (GI) [5,6,7] came into being in 1995. As an alternative of SPI, it records the total light intensities during each modulation, and calculates the intensity correlation between modulation patterns and detected bucket signals to acquire a 2D image. Entering the new century, it usually utilizes pseudo-thermal random-modulated patterns. However, to obtain a nice reconstruction, the statistical mechanism of GI requires that the number of measurements is much higher than *N*. Hadamard [8,9,10] and Fourier [11,12] SPI are two other computational imaging techniques that use complete deterministic orthogonal bases to sample the scene, allowing one to reduce the number of measurements back to *N*. However, all above approaches still involve a large amount of signal acquisitions, accompanied by the questions of transmission and storage.

Recently, many SPI methods based on compressed sensing (CS) [13,14,15] have been proposed to acquire a better imaging performance with subsampling ratios (from much fewer than *N* measurements) by exploiting the sparsity of the object expressed in a certain basis, at a cost of increased computational time, especially for large-scale images. For general cases, the number of measurements M∼4αlog(1/α)N is enough for retrieving a high-quality reconstruction [16], where α stands for the sparsity ratio of the object image. We note that the larger the dimensionality of the image, the smaller α becomes. The sampling ratio will be accordingly smaller with larger pixel resolution, especially for megapixel images. However, for more modest resolutions, one can hardly acquire a good image quality with traditional CS when the sampling ratio is below 30%. The main reason is that the measurement matrix used by CS is generally random and the sampling process is relatively blind, thus there still exists redundancy in the measurements. Moreover, the higher is the pixel resolution, the more stringent are the computational restrictions. As we know, many CS applications in fields including magnetic resonance imaging [17], astronomy [18], and microscopic imaging [19], have the resolution limitation (generally smaller than or equal to 128×128 pixels) caused by the sampling rate.

To further reduce the sampling rate, one effective way is to remove the redundancy in the random measurements. Intuitively, using patterns that are more similar or have higher correlations with the object may have a higher reconstruction contribution with lower sampling ratios, i.e., make as many modulation spots as possible fall on the object part. However, it requires us to know the outline of the object in advance and to make the shape of the patterns change with target object, which is actually unrealistic. A study on correspondence imaging (CI), first proposed by Luo et al. [20,21] in 2011, shows that the measured values can be used as weights to indicate whether their corresponding patterns are positively or negatively correlated with the object to be detected. It arranges the measured values from large to small, with the patterns corresponding to the largest detected values being computed first. The correspondence images can be retrieved from considerably fewer frames of patterns by only calculating the average of the patterns that correspond to the measured values much larger than (or less than) the mean. The essence of this interesting phenomenon was firstly answered in an attempt at some theoretical explanations [22,23,24,25], and lately was strictly explained by using a probability theory, which treats light intensities as stochastic variables and deduces the joint probability density function, followed by an analysis of the visibility and contrast-to-noise ratio (CNR) vs. the threshold parameter (i.e., former percentage) and the unit number of the object [26]. Diverse CI-based schemes [27,28,29] have been developed to promote the practical use of CI technology. Its shortcoming is that we should perform the whole sampling before computing the accurate mean value. Recently, applying deterministic Hadamard full sampling rather than random sampling, a technology named evolutionary compressive sensing (ECS) [8,9] was developed for SPI, where the patterns are chosen based on the prior knowledge of the estimated mean from the first few acquisitions, without post-processing. In fact, its principle is the same as the sort strategy of CI. ECS asserts that utilizing this sort strategy attains the fraction of patterns with the largest contributions to the image reconstruction [9]. More recently, an alternative approach called “Russian Dolls” (RD) ordering [30] of the deterministic Hadamard basis is proposed to further demonstrate this idea. It uses four quarters in each layer and finally reorders the patterns within each quarter. This means that RD can yield a comparable quality to CS, but with limited spatial resolution (only 128×128 pixels) due to high computational complexity of sorting. Both CI and ECS provide us with a good inspiration but still incur a trade-off between reconstruction quality and real-time robustness.

Here, we present an alternative practical method that can acquire high-quality images of large pixel scale with super sub-Nyquist sampling ratios, by using a strategy that we call the “cake-cutting” (CC) sort to optimally reorder the Hadamard basis, based on the contribution of the pattern basis to the reconstruction. Following this idea, our method offers a new way without any prior average processing to always make the most significant patterns be modulated firstly. Meanwhile, by utilizing the structured characteristic of Hadamard matrix, the computational overhead and the memory consumption is also greatly reduced. It is shown through numerical simulation to significantly reduce the number of measurements along with the acquisition time. Furthermore, with a single-photon single-pixel camera setup based on differential modulation, we have demonstrated its ability to retrieve clear images even through partially obscuring scenes under noisy environmental illumination conditions.

## 2. Theory and Methods

### 2.1. Principles Description

The core idea of SPI is to transfer the spatial resolution from the array detectors to the modulated patterns to acquire the spatial information of the target. These patterns are of the same spatial resolution p×q=N with the target image *x*, while the latter can be reshaped into a column vector of size N×1. By leveraging the fact that natural images can be sparsely represented in an appropriate basis Ψ, i.e., $x=\Psi x^{\prime }$, compressive imaging methods allow one to reconstruct the images from M=O(K·log(N/K))<N random patterns, considering all but the largest K(≤N) elements of the sparse representation coefficients in a certain basis to be set to zero. Here, α=K/N is the sparsity ratio, and Ψ is an invertible (e.g., orthogonal) matrix or a redundant dictionary. We define the ratio M/N as the sampling rate. Thus, CS in principle provides a benefit in reducing acquisition time. There are many ways of performing compressive sampling, such as digital micromirror device (DMD) modulation, Fourier filtering, etc. In this work, the patterns are modulated by a DMD. It consists of millions of micromirrors, each of which is orientated either $12^{\circ }$ or $-12^{\circ }$ with respect to the normal of the DMD work plane, corresponding to the bright pixel 1 or the dark pixel 0. Each pattern $a_{ij}$ sequentially encoded on the DMD can be flattened into a row vector of size 1×N, thus *M* such binary patterns constitute a known M×N measurement (or sensing) matrix *A*. This measurement matrix actually projects the object signal *x* into a single-pixel (bucket) compressed signal y=Ax+e of smaller size M×1. Here, *e* of the same size M×1 denotes the stochastic noise. Thereby, the single-pixel total intensity measurement is mathematically equivalent to the inner product between each pattern and the object image, i.e., the interaction between the pattern sequence and the object scene. Then, the goal is to solve an ill-posed linear problem through optimization algorithms by finding the sparsest representation $x^{\prime }$ such that $y=A\Psi x^{\prime }+e$. Here, we apply the total variation minimization (TVAL3 solver) [31], which can be written as an augmented Lagrangian function:

(1) $$\min_{x}\sum_{i}\left|\right|D_{i}{x||}_{p}+\frac{\mu }{2}||y-{Ax||}_{2}^{2},$$

where ${\left||x|\right|}_{p}=(\sum_{i=1}^{N}|x_{i}{{|}^{p})}^{\frac{1}{p}}$, $D_{i}x$ denotes the discrete gradient vector of *x* at the *i*th position, *D* is the gradient operator, and μ is a balance constant.

To ensure a good estimation of $x^{\prime }$, the measurement matrix *A* should satisfy the restricted isometry property (RIP) [32,33], which requires the sensing matrix with the property that column vectors taken from arbitrary subsets are approximately orthogonal and incoherent with Ψ. As we know, the Hadamard matrices fulfill this property and can serve as the sensing matrix. A Hadamard matrix [34], named after the French mathematician Jacques Salomon Hadamard, is a symmetric square matrix with entries ±1. Now, let *H* be a Hadamard matrix of dimension *N*, where *N* is a power of two. Then, we have $HH^{T}=NI_{N}$ and $H^{T}=H$, where $I_{N}$ is the N×N identity matrix and *T* represents the transpose operator. Namely, dividing *H* by $\sqrt{N}$ gives an orthogonal matrix whose transpose equals its inverse. The naturally ordered Hadamard matrix of order $2\leq 2^{k}\in N$ can be given by the following recursive formula

(2) $$H_{2^{k}}=\left[\begin{array}{cc} H_{2^{k-1}} & H_{2^{k-1}}\\ H_{2^{k-1}} & -H_{2^{k-1}} \end{array}\right]=H_{2}⊗H_{2^{k-1}},$$

where $H_{1}=\left[1\right]$, $H_{2}=\left[\begin{array}{cc} 1 & 1\\ 1 & -1 \end{array}\right]$, ⊗ stands for the Kronecker product. It is interesting to note that the elements in the first row and the first column are all ones. The sequency-ordered (also known as Walsh-ordered, named after an American mathematician Joseph Leonard Walsh) Hadamard matrix can be easily formed by rearranging the rows of the above matrix, according to an increasing order of the number of sign changes of each row. The detailed permutation steps are the bit-reversal permutation followed by the Gray-code permutation. Other variants of Hadamard matrix also exist, such as dyadic-ordered Hadamard matrix. All of the above forms can be easily converted into each other.

If we choose a matrix with the entries either +1 or −1 as the measurement matrix *A*, then the bucket signal y=Ax will also present a positive–negative distribution. Generally, the front few fractions of the subset of *A* with respect to $|y_{i}|\gg \langle |y|\rangle$ will yield a good image quality because these patterns have a significant effect on image reconstruction, where 〈·〉 signifies the ensemble average. This method is called sorted compressed sensing (SCS).

**Figure 1:**
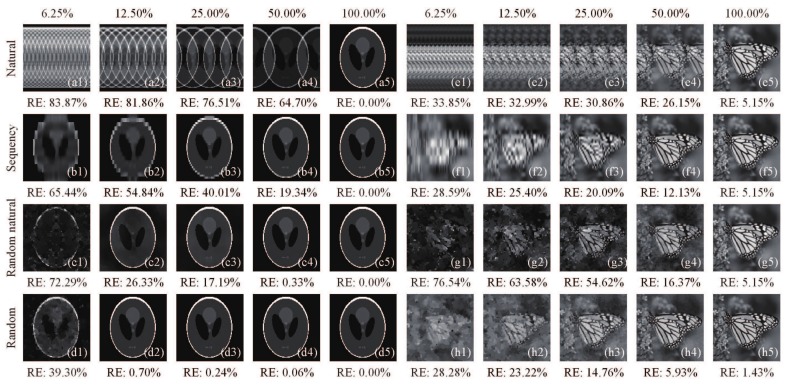
Reconstructed images of head phantom image and monarch butterfly image (with more complicated structure) from: (**a1**–**a5**,**e1**–**e5**) natural-ordered; (**b1**–**b5**,**f1**–**f5**) sequency-ordered; (**c1**–**c5**,**g1**–**g5**) random-natural-ordered Hadamard matrix; and (**d1**–**d5**,**h1**–**h5**) totally random measurement matrix with different sampling ratios of 6.25%, 12.50%, 25.00%, 50.00% and 100%. Note that the random-natural-ordered Hadamard matrix only randomly rearranges the rows of the natural-ordered Hadamard matrix.

It can be seen from the above method that the intensity fluctuations of the single-pixel (bucket) signal play an important role in acquiring high quality images, and the most significant patterns should be modulated first. Although there exists many orderings for the Hadamard matrix, as mentioned above, none of them can make the most significant patterns appear first. Here, we give a comparison of recovered results using natural-ordered, sequency-ordered, random-natural-ordered Hadamard (RH) matrix (where only the row order of the natural-ordered Hadamard matrix is random), and totally random measurement matrix with different sampling ratios of 6.25%, 12.50%, 25.00%, 50.00% and 100%, as shown in Figure 1. To obtain a quantitative measure of the image quality, the relative error (RE) is defined here as a figure of merit:

(3) $$\mathrm{RE}=\frac{\left|\right|\tilde{U}-U_{o}{\left|\right|}_{F}}{\left|\right|U_{o}{\left|\right|}_{F}}\times 100\%,$$

where $\tilde{U}$ denotes the reconstructed image and $U_{o}$ stands for the original image, all of p×q pixels. Here, ${\left||X|\right|}_{F}$ is called Frobenius norm which can be defined as

(4) $${\left||X|\right|}_{F}=\sqrt{\sum_{i=1}^{p}\sum_{j=1}^{q}{\left|X_{ij}\right|}^{2}}=\sqrt{\mathrm{trace}(X^{*}X)}=\sqrt{\sum_{i=1}^{min\{p,q\}}\sigma_{i}^{2}},$$

where * denotes the conjugate transpose operator, and $\sigma_{i}$ is the singular value of *X*.

We can see in Figure 1 that directly using the natural-ordered Hadamard matrix, the reconstructed image will have a periodic silhouette ghosting, which is caused by the structural features of the matrix itself. The sequency-ordered Hadamard matrix will also have a ghosting on the image detail at low sampling rates. Randomly rearranging the rows of the natural-ordered Hadamard matrix can greatly reduce the correlation between the patterns, but its performance is not as good as that of pure random measurement matrix. When the sampling ratio equals 100%, i.e., full sampling, the performance of natural-ordered, sequency-ordered and random-natural-ordered Hadamard matrix will be the same. Although the image can be efficiently retrieved from fewer measurements sampled by the random measurement matrix, random measurements still have measurement redundancy, and suffer from high computation cost, vast storage and uncertain reconstruction qualities. Besides, it is hard to know a priori which patterns can generate the most significant intensity values; one can only pick up the crucial patterns needed according to the recorded signal after the sampling is completed. To our knowledge, some definite matrices such as Toeplitz matrix [35,36] and polynomial matrix [37] are other common choices, which can reduce the computational overhead and are much easier to be implemented, but with poorer reconstruction qualities than that with a random matrix. Therefore, designing a sampling matrix that makes the most significant patterns modulate first becomes very important; it will undoubtedly lead to a better reconstruction result and a smaller sampling ratio than the random matrix. The above rearrangements of the Hadamard basis offers the possibility of such kind of optimal modulation.

Recently, the RD ordering [30] of the Hadamard basis provides us with some inspiration. The rows of the Hadamard matrix are ordered such that the top half of $H_{2^{2z}}$ is equal to the rows of $H_{2^{2z-1}}$ (in the two-dimensional pattern view, the former is a scaled version of the latter with a factor 2), similar to a Russian dolls set, then the third quarter of $H_{2^{2z}}$ are ordered as the transpose of its second quarter, the rest are catalogued into the fourth quarter, and at last the patterns within each quarter are reordered again. By this means, the image can be reconstructed from a subset (e.g., 6% sampling ratio) of significant patterns, with a quality comparable to that of CS. There is no need to disorder internal pixel layout in each pattern. However, the RE of RD-based GI method presents a sawtooth descent as the sampling ratio increases, so that its performance curve is neither stable nor smooth. Since this method is based on the second-order correlation, it is sensitive to the environmental noise. Additionally, the pixel resolution is also limited, generally 128×128 pixels, as this ordering operation is too complex. Meanwhile, the sampling ratio is still limited, causing a long acquisition time especially for imaging at large pixel resolution.

### 2.2. Cake-Cutting Hadamard Basis Sort

Actually, the bucket intensities can be regarded as stochastic variables (the weight). The visibility as well as the CNR of recovered images versus the pattern selection (threshold parameter) and the unit number of object has already been strictly explained via a joint probability density function between the bucket and reference signals [26]. The CNR decreases when the object image becomes dense, and the CNRs of correspondence images will be optimal when the bucket threshold is the ensemble average. This theory well explains that partial patterns have a positive contribution to image reconstruction while others only have a negative or averaged contribution to image reconstruction.

Furthermore, as described in differential ghost imaging (DGI) [7], assume that the light field distribution of the source and the object beam are *O* and $O_{b}$, respectively, the number of independent measurements is *m*, the (intensity) transmission function of the object is denoted by *T*, and the minimum variation of the object transmission function is indicated as $\Delta T_{\min}$. Then, the signal-to-noise ratio (SNR) of GI methods can be written as

(5) $${\mathrm{SNR}}_{\mathrm{GI}}=\frac{{\left[\Delta \langle O_{b}\rangle \right]}_{\min}^{2}}{\langle \Delta O^{2}\rangle }=\frac{m}{N_{\mathrm{speckle}}}\frac{\Delta T_{\min}^{2}}{\bar{T^{2}}},$$

where $N_{\mathrm{speckle}}=A_{\mathrm{beam}}/A_{\mathrm{coh}}$ is the number of speckles in the beam and $A_{\mathrm{beam}}$ is the area of the light beam. Set the size of each speckle to be $\delta_{0}$, which can be determined by the Hanbury–Brown–Twiss (HBT) algorithm, i.e., compute the autocorrelation half height width of each point of the light field, then we will have $A_{\mathrm{coh}}=\delta_{0}^{2}$. From this formula, it is much easier to recover an absorbing object ($\bar{T^{2}}\ll 1$) than a quasitransparent object ($\bar{T^{2}}∼1$). When the object is fixed, the term $\Delta T_{\min}^{2}/\bar{T^{2}}$ is also fixed, and then this formula can be simplified as ${\mathrm{SNR}}_{\mathrm{GI}}\propto \frac{m}{N_{\mathrm{speckle}}}$. This also explains why different sorting methods lead to the quality change of compressive imaging: the reordering of the measurement basis may cause the change of coherence area of each speckle. Only when the number of measurements increases and the coherence area changes from large to small, the SNR of the reconstruction will rises rapidly. We find that, if the number of connected domains of the Hadamard basis is sorted in an ascend order, the coherence area of each speckle will be naturally changed from large to small. By this means, we can generate an optimized sort of the Hadamard basis and obtain the best results with much smaller sampling ratios.

In this work, each row of the Hadamard matrix $H\in R^{N\times N}$ is reshaped into a matrix of p×q=N pixels. Imagine each reshaped basis pattern as a cake; we can count how many pieces this cake is cut into, and each piece can be regarded as a speckle (a connected region). Thus, this method is also named as a cake-cutting (CC) strategy. In topology and mathematics, a connected region is a topological region that cannot be represented as the union of two or more disjoint nonempty subsets. This suggests the values in each piece of the cake are either all −1s (in black) or 1s (in white). Thereby, the piece number of the cake can be denoted by the number of connected regions of −1 plus that of 1. Besides, for one pixel in one basis pattern, its adjacent pixels (up and down, left and right) with the same values can be all treated as one part of its connected region. According to the CI theory described above, only a small fraction of the complete patterns contributes to a larger intensity value $|y_{i}|$. Here, we find that the fewer connected regions a basis pattern contains, the greater the probability of this pattern to be significant or to generate a higher measured value for a same object. Therefore, we order the complete Hadamard basis patterns according to their piece numbers and acquire a sort sequence seq of size N×1. After that, the *N* reordered patterns can be flattened into *N* row vectors, each of size 1×N, forming a N×N measurement matrix.

**Figure 2:**
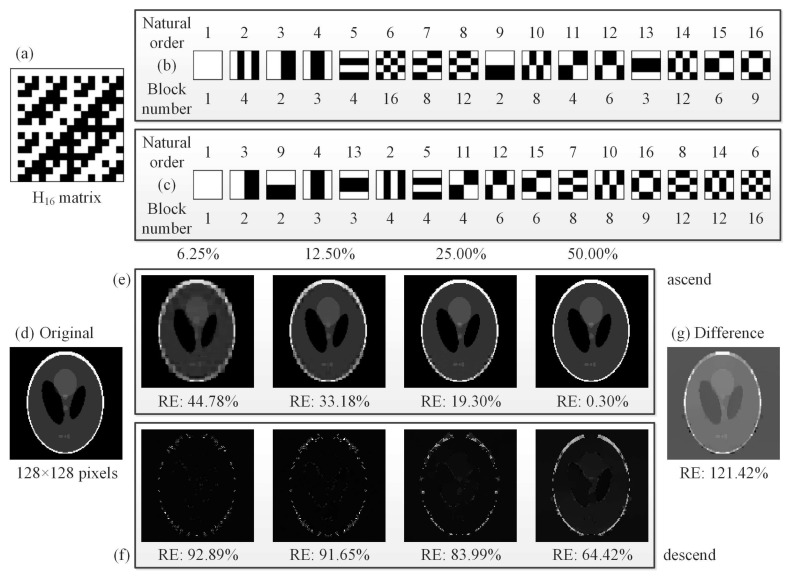
An example for the “cake-cutting” Hadamard basis: (**a**) a 16×16 Hadamard matrix $H_{16}$; (**b**) the basis patterns of $H_{16}$ in the natural order; (**c**) the basis patterns of $H_{16}$ in our optimized “cake-cutting” order; (**d**) an original head phantom image of 128×128 pixels; (**e**,**f**) comparison of CS reconstructions with the first 6.25%, 12.50%, 25.00% and 50.00% of the fully sampled measurements while the piece numbers are in their ascending order and descending order, respectively; and (**g**) the difference image of the fourth images of (**e**,**f**).

Here, Figure 2 gives an example how our cake-cutting Hadamard basis sorting works. By picking out each row of the $H_{16}$ matrix (Figure 2a) and transforming each row into a 4×4 2D pattern, a complete set of 16 Hadamard basis patterns is then presented in Figure 2b, e.g., in the natural order. Without loss of generality, here the original order can also be other order sequence, such as sequency order or dyadic order. Following our cake-cutting strategy, these Hadamard basis patterns are sorted by their piece numbers (see Figure 2c).

Next, we generate a Hadamard matrix $H_{16,384}$ of order N=128×128, which is used to reconstruct an object image of 128×128 pixels. By using this method, we compare the performance of recovered images from the front 6.25%, 12.50%, 25.00% and 50.00% measurements when the piece (block) number of each pattern is in ascending order and descending order, respectively, as shown in Figure 2e,f. From the RE data, we can see that the performance is in proportion to the compression ratio for the ascending order of piece numbers but this feature is not suitable for the descending order. It is easy to understand because a basis with larger piece (block) number can be regarded as a totally random pattern to some extent (more likely to generate an averaged single-pixel value), which will undoubtedly degrade image quality or weaken imaging efficiency. One may think that the difference of the corresponding recovered images will produce a better quality, but it does not turn out that way due to the environmental noise, as shown in Figure 2g.

### 2.3. Fast Hadamard Computation

There are many fast CS algorithms based on different principles for imaging at large-scale resolution, such as RecPF algorithm based on a partial Fourier matrix and NESTA algorithm based on an orthogonal projector. They do not apply randomized Hadamard matrices and can reconstruct megapixel-sized images even with a laptop computer with small random access memory. Since the sampling matrix used here is generated from the Hadamard matrix, we apply TVAL3 algorithm [31] in this work. The original TVAL3 algorithm uses the Kronecker product and recursive formula to perform fast Walsh–Hadamard transform (FWHT), which was implemented in C++ and then compiled and linked into a shared library called a binary MEX-file in MATLAB software. By running FWHT, only additions and subtractions are involved instead of matrix multiplications. Thus, the FWHT implementation is an efficient algorithm which can reduce the computational complexity of original *n*-order Walsh–Hadamard transform (WHT) from $O(n^{2})$ to O(nlogn), where *O* is short for the order. By this means, the storage of matrix is unnecessary. Note that the Kronecker product works on the natural-order Hadamard matrix, thus the sequency-ordered Walsh–Hadamard transform is obtained by first performing FWHT, and then rearranging the outputs by bit-reverse and Gray code conversion. Notice that the original TVAL3 software needs to randomly permute both columns and rows of the Hadamard matrix and to select *M* rows to form an incoherent and totally random binary M×N matrix with entries either 1 or −1. However, to well combine the CC ordering with FWHT in this work, we have to rewrite the FWHT function through the m-file in MATLAB (according to classic thoughts, another approach with a similar performance, which is much easier to reproduce), rather than using TVAL3 MEX-library-file (read-only and non-editable) or MATLAB built-in function fwht and its inverse function ifwht (very slow). Additionally, another difference in our CC method is that only the rows of the Hadamard matrix need to be rearranged, while the order of the columns of original Hadamard matrix remains unchanged.

**Figure 3:**
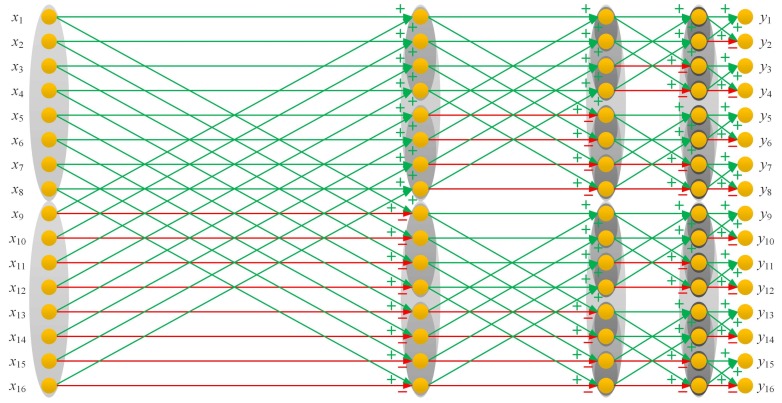
Simplified mathematical model (graph) for the Hadamard matrix multiplication calculation, taking $H_{16}$ for an example.

The FWHT algorithm we used can be treated as a divide and conquer algorithm taking full advantage of the structured characteristic of the Hadamard matrix, which recursively divides a WHT problem of size *n* into two smaller WHT sub-problems of size n/2[38]. The idea of the FWHT applied to a column vector of length 16 is illustrated in Figure 3. Actually, the operation $H_{N}x=y$ can also be explained by a graph with a set of vertices of edges. The weight of each edge is either 1 or −1. Similar to neural networks or convolutional neural networks, the Hadamard matrix can be regarded as a propagation function or a network consisting of connections, in which each connection transfers the output of a neuron *i* to the input of another neuron *j*, whilst $x={\left\{x_{1},x_{2},x_{3},\ldots ,x_{N}\right\}}^{T}$ and $y={\left\{y_{1},y_{2},y_{3},\ldots ,y_{N}\right\}}^{T}$ can be treated as the original input and the final output. There should be $\log_{2}N-1$ hidden layers, depending on the order *N* of the Hadamard matrix. Additionally, there are *N* transverse edges and *N* intersection edges from the current layer to the next layer, and the number of the neurons in each layer is all *N*. Form the graph, it is interesting to find that all oblique intersection lines are in green, and all the lines that point to the right are half in green and half in red, where green stands for plus and red represents minus.

For the Hadamard matrix *H* of order *N*, the number of the computation layers for the fast computation of the formula $H_{N}x=y$ should be $\log_{2}N$. First, we initialize an intermediate column vector *b* such that b=x and let t=N/2 be the original length of each group in the first layer (i.e., the input layer). For the *i*th layer, expect for the output layer, i.e., i=1,2,3,…logN, the current *x* can be divided into $2^{i}$ groups. In the *i*th layer, we will traverse $2^{i-1}$ times to compute every element in each group such that temp=b(index), b(index)=temp+b(index+t) and b(index+t)=temp−b(index+t), where the range of index is from 1+2(j−1)t to (2j−1)t, *j* is from 1 to $2^{i-1}$, and *t* denotes the length of each group in this layer. In the next (i+1)th layer, we update *t* via t=t/2, repeat the above operations until the $\left(\log_{2}N+1\right)$th layer is reached. At last, y=b. If we want to pick out the $r_{i}$th row of the Hadamard matrix to form a modulated pattern, it only needs to compute $H_{N}x$ where the $r_{i}$th element of *x* is set to one with the rest elements of *x* all zeros. The operation $H_{N}^{-1}x$ is equivalent to calculating $\frac{1}{\sqrt{N}}H_{N}^{T}x=\frac{1}{\sqrt{N}}H_{N}x$. Similarly, the $r_{i}$th element of *y* can also be easily obtained after performing the above graph calculation. As mentioned above, to obtain the sequency-ordered FWHT, we only need to rearrange the outputs. Here, we perform our cake-cutting strategy on the sequency-ordered operator for illustration. We choose the front *M* elements of the output signal *y* according to the cake-cutting sort sequence.

**Figure 4:**
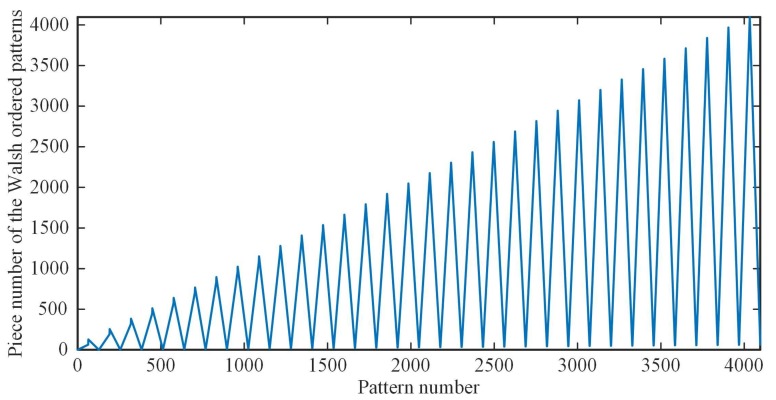
The piece number of the sequency-ordered patterns as a function of the pattern number.

As for the order sequence generation time of our method, we present the comparison results in Table 1. Here, we hypothesize that the image to be reconstructed is square, i.e., p=q. Since there is no need for the nested grouping like in RD method, our approach can greatly reduce the sequence generation time, especially for the large scale Hadamard matrix. However, we still think that computing the number of the connected regions is time consuming (see the CC row in Table 1), thus we plot the curve of the piece number of the sequency-ordered patterns as a function of the pattern number, as shown in Figure 4. Fortunately, we find that there exists a remarkable regularity for i=1,2,3,…,q, that is

(6) $$\left\{\begin{array}{c} Seq\left[(i-1)p+1:ip\right]=i:-{\left(-1\right)}^{mod(i,2)}i:ip,\;\mathrm{for}\;i\;\mathrm{is}\;\mathrm{odd},\\ Seq\left[(i-1)p+1:ip\right]=ip:-{\left(-1\right)}^{mod(i,2)}i:i,\;\mathrm{for}\;i\;\mathrm{is}\;\mathrm{even}. \end{array}\right.$$

Then, we can rearrange the piece numbers in their ascending order to obtain the CC sequence. The order sequence generation time of ${\mathrm{CC}}_{\mathrm{rule}}$ for different *n* is given in the fifth row of Table 1. It can be seen that this rule can further reduce the generation time of our CC method, breaks both computational and memory limits, and makes our method more practical. Note that this regularity is only effective for the sequency-ordered patterns. If we choose a different ordered (such as natural-ordered or dyadic-ordered) operator as the initial order of the FWHT, we should accordingly adjust the recursion formula, otherwise the optimized order will be incorrect. Actually, all computation of these orders needs to be performed only once, after which we can simply use the ordered generated by this calculation, thus the length of sequence generation time is less important and not the key point of this work, but it does bring benefits as above.

Now let us explore the reasons of the above rule more deeply. The term “sequency” can be used to designate the number of sign changes of Hadamard basis patterns. Then, the sequency-ordered Hadamard matrix can be generated by making the rows of natural-ordered Hadamard matrix be sorted in a form in which the sequency of each row is larger than its preceding row [34]. Starting from a natural-ordered Hadamard matrix, the number of one-dimensional connected domains of its each row is exactly equal to the number of sign changes plus 1. When this basis pattern is reshaped into a 2D matrix, we can denote the number of row sign changes and that of column sign changes as ${\mathrm{Sequency}}_{r}$ and ${\mathrm{Sequency}}_{c}$, respectively. Then, the number of pieces of cakes (also termed connected regions) is exactly equal to $\left({\mathrm{Sequency}}_{r}+1\right)\times \left({\mathrm{Sequency}}_{c}+1\right)$, due to the strong structural characteristics exhibited by the Hadamard matrix. If traversing the sequency-ordered (or natural-ordered) Hadamard basis patterns, we can derive the same sequence of patterns as that derived by above rule. In other words, it is the structural characteristics of sequency-ordered Hadamard matrix that create the oscillating rising waveform of the number of its 2D connected domains of patterns.

Actually, any patterns with small piece (block) number can improve the imaging efficiency, i.e., we can start from any initial patterns and control their block numbers as well as the ratios of ±1 in these patterns. For example, we can first select the largest square pixel unit, which contains multiple pixels, to form a random pattern sequence, and then rearrange these patterns with their piece (block) number in a descending order. Next, we can choose smaller square pixel unit followed by the same process, and augment the new patterns into the above sequence. This approach offers an alternative way to generate an optimal CC pattern sequence, but it will consume a large amount of memory and computing resources when reconstructing a large-scale image. Moreover, the computation will become too complicated for an ordinary computer to bear. However, choosing the Hadamard basis as the initial pattern sequence, we can make full use of the structural features of the Hadamard matrix to simplify matrix multiplication and reduce memory consumption. Therefore, the Hadamard basis is necessary for our CC method.

## 3. Results

### 3.1. Numerical Simulations

**Figure 5:**
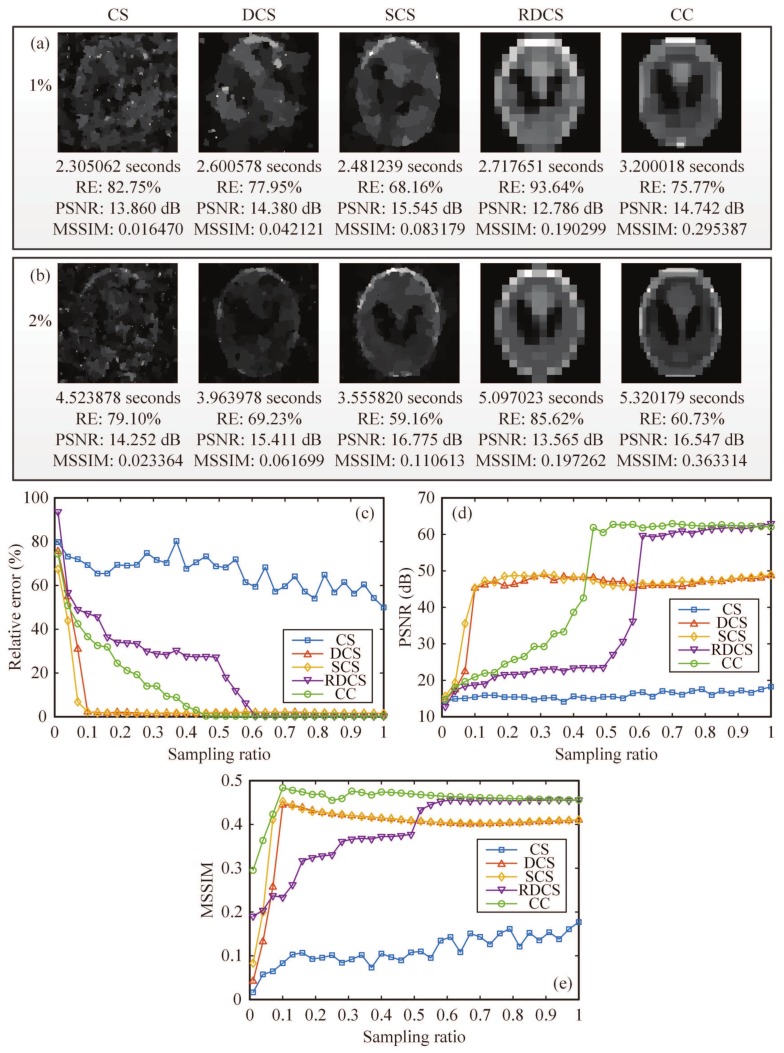
Comparison of the simulation results: (**a**,**b**) The reconstructions using CS, DCS, SCS, RDCS, CC method, with a sampling rate of 1% and 2%, respectively. The corresponding evaluation parameters such as the recovery time, relative error (RE) and peak signal-to-noise ratio (PSNR) are also given here. The recovery time can be shorter with some faster computers. (**c**–**e**) The comparison of above methods, in terms of RE, PSNR and MSSIM as a function of the sampling ratio. All images are of 128×128 pixels.

**Figure 6:**
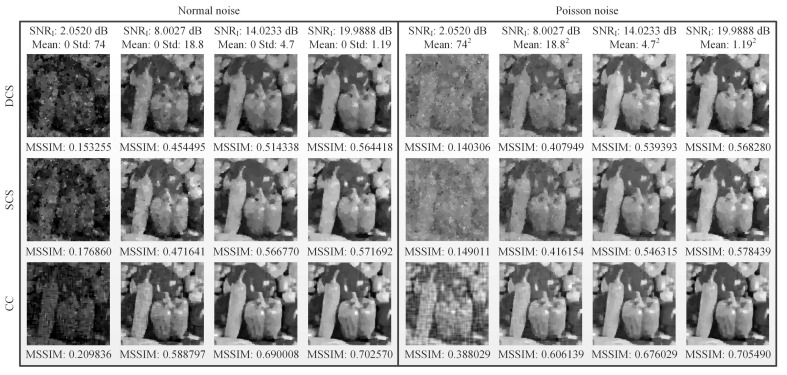
Influence of different noise on the image reconstructions, using DCS, SCS and CC methods. Two kinds of illumination fluctuation noise are set, one following normal distribution, and the other obeying Poisson distribution. All recovered images are of 128×128 pixels, with a 12.5% sampling ratio.

**Figure 7:**
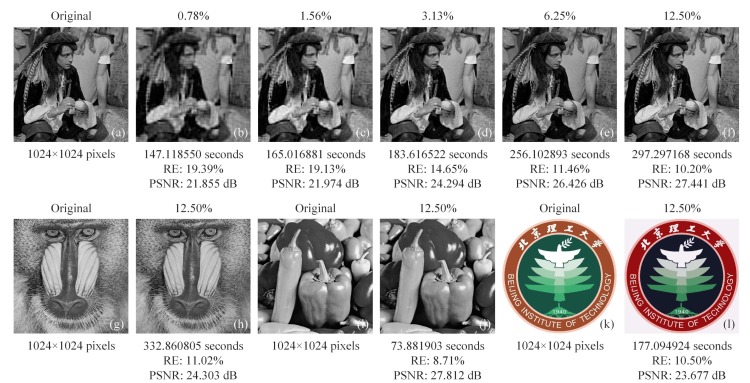
Reconstructions of different 2D images, covering gray-scale and color-scale, all of 1024×1024 pixels: (**a**) an original man image; (**b**–**f**) retrieved images with a 2× stepping increase of the sampling ratio; (**g**) an original mandrill image; (**h**) recovered image of the mandrill with a sampling ratio of 12.50%; (**i**) an original image of peppers; (**j**) the reconstructed image of peppers using 12.50% measurements; (**k**) the original color image of the school badge of Beijing Institute of Technology; and (**l**) the recovery of the school badge with a compression ratio of 12.50%. All original images are open access. We acknowledge Beijing Institute of Technology for the permission of using the school badge as an object.

**Figure 8:**
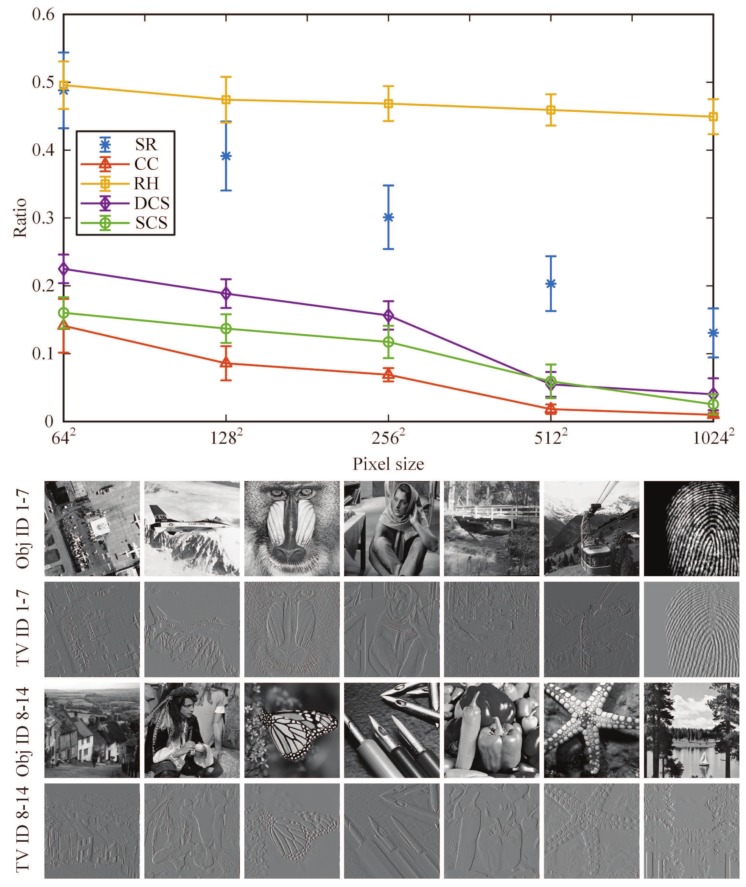
Sampling ratios of CC, RH, DCS, and SCS methods compared with the sparsity ratio as a function of image size, under a number of different object samples (of Obj IDs 1–14), with a PSNR around 20.334 dB (in which the detail of the object just can be resolved). We add the error bar to each point and the height of each error bar indicates the standard deviation of each point. In the bottom, 14 object samples used and their TV graphs are presented.

**Figure 9:**
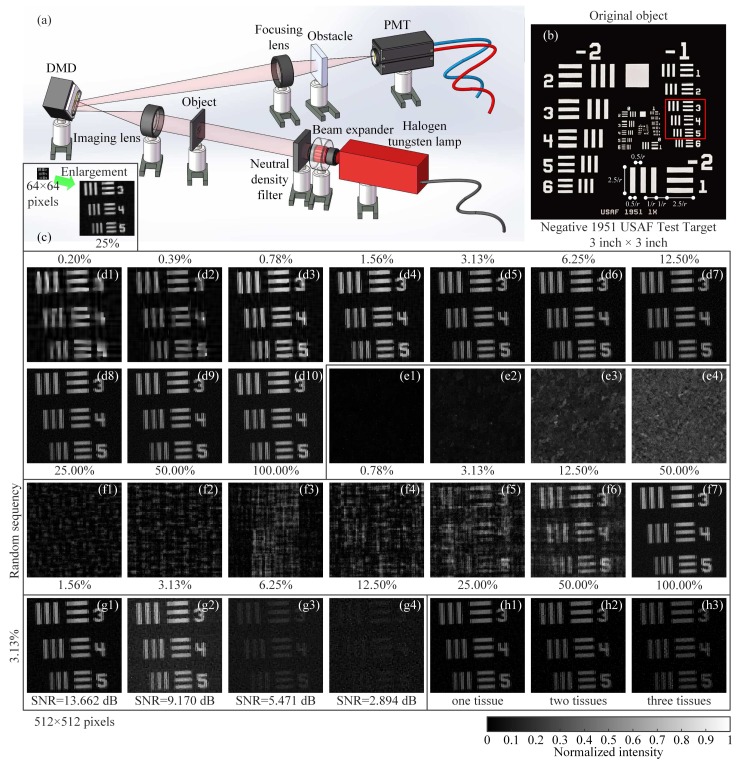
Schematic of the optical setup and experimental results. (**a**) The thermal light from a Halogen tungsten lamp illuminates the object through a beam expander and some NDFs, and then the light is projected onto a DMD. The reflected light is collected by a PMT through a focusing lens. (**b**) A negative 1951 USAF resolution test chart of 3 inch × 3 inch is treated as an object to be detected. (**c**) The recovered image of 64×64 pixel size with a sampling ratio of 25%. (**d1**–**d10**) Reconstructed images of 512×512 pixels using the sampling ratios from 0.2% (the limit) to 100% with a 2× stepping increase, when the piece numbers are in their ascending order. (**e1**–**e4**) Retrieved images of 512×512 pixels with a 4× stepping increase in the sampling ratio, when the piece numbers are in their descending order. (**f1**–**f7**) For comparison, the recovered images using traditional random-natural-ordered Hadamard sensing matrix are given, with the sampling ratios changing from 1.56% to 100.00% with a 2× stepping increase. (**g1**–**g4**) The reconstructions via our method under different SNRs, acquired without obstacles. (**h1**–**h3**) The results using our method with obstacles (some tissues, the number of which is increasing). The results in the last row all use a sampling ratio of 3.13%.

To test the performance of our method for image reconstruction, some numerical simulations were performed. Here, we introduce another unitless performance measure, the peak signal-to-noise ratio (PSNR), which is defined as

(7) $$\mathrm{PSNR}=10\log(255^{2}/\mathrm{MSE}),$$

where $\mathrm{MSE}=\frac{1}{pq}\sum_{i,j=1}^{p,q}{\left[U_{o}\left(i,j\right)-\tilde{U}\left(i,j\right)\right]}^{2}$. The MSE describes the squared distance between the recovered image and the original image. Naturally, the larger is the PSNR value, the better is the quality of the image recovered. To allow a fair comparison of the image quality, all the recovered images in Figure 1, Figure 2 and Figure 5, Figure 6, Figure 7 and Figure 8 are normalized to a range of 0∼255. Since the optical experiments generally have no original image as a reference, the experimental results in Figure 9 are directly normalized to a range of 0∼1.

Then, we also used a head phantom image as the original image. The reconstructed results were acquired from 1% and 2% measurements by using five different approaches: CS, differential compressed sensing (DCS), SCS, “Russian Dolls” CS (RDCS), and our CC method (Figure 5a,b). It is worth mentioning that here the “Russian Dolls” method is applied to compressive imaging, rather than GI in its original scheme [30], definitely generating a better image quality. In the simulation, compared with the other existing methods, it only takes a little more (negligible) time for our CC method to iteratively compute the images, but yielding a much better performance. Then, we drew the RE and the peak signal-to-noise ratio (PSNR) of reconstructed images as a function of the sampling ratio. In Figure 5c,d, it is clearly seen that our CC method is much better than the CS and RD methods with an overwhelming superiority for any sampling ratio. In fact, both RDCS and CC are the sorting method based on the Hadamard basis, and the front patterns with larger piece numbers form the low frequency components of the target, while the image quality will increase when we continuously add the patterns with larger piece numbers. When the sampling ratio is over 50%, the PSNR of RDCS presents a rapid change; it is easy to understand because the Hadamard matrix has a very good structure. According the principle of RDCS, when the sampling ratio exceeds 50%, the patterns with larger piece numbers will be modulated with a higher probability. From the PSNR curve, it can be seen that CC will exceed the DCS and SCS methods when the sampling ratio is over 40%. As mentioned above, SCS has a major drawback that it needs to fully sample the image, and then to pick up the most significant intensities. Our CC method makes up for this defect. It is important to note that, when the sampling ratio is below 40%, it seems that our proposed method shows no advantages to DCS and SCS. That is because the RE and the PSNR, serving as the performance metrics, all quantify the visibility via the calculation of pixel errors. These pixel-wise performance measures may fail to capture the structure of natural images and may cause evaluation misjudgments; i.e., an image which is supposed to have a better visibility may instead have a worse RE or PSNR value. Actually, in Figure 5a,b, the CC method has a much better visibility than those of other four methods when the sampling ratio is very low, i.e., 1% and 2%, but cannot be characterized very well with the RE or PSNR data of Figure 5c,d. These examples illustrate the weak correlation of RE and PSNR with perceptual quality under some special cases, and that they sometimes cannot effectively tell the reconstruction quality. The image contrast, brightness and pixel perturbations all will affect the accuracy of PSNR and RE values. Thus, here we introduce another quantitative index mean structural similarity (MSSIM) [39], which is a full reference metric for evaluating the perceptual difference between two similar images (a reference and a processed image). Unlike RE or PSNR, MSSIM is mainly based on visible structures in the image, with a value range between 0 and 1. The larger is the MSSIM value, the better is the image quality. When MSSIM=1, the processed image is identical to the reference image. The corresponding MSSIM curve is given in Figure 5e, and the MSSIM values for Figure 5a,b are also provided. From the MSSIM values, we can see that the MSSIM values of CC are always better than those of other four methods, consistent with the visible results of Figure 5a,b, while the MSSIM values of RDCS present a ladder rising trend, which keeps in accordance with the theory of RDCS. When the sampling ratio is large enough, the MSSIM values tend to saturate. Therefore, the image quality does not increase indefinitely as the sampling ratio increases.

Generally, there are many noise sources involved in the imaging process, such as illumination fluctuation noise, detector noise, etc. In mathematical expressions, all of them can be finally converted to an additive noise, thus their effects are similar. Here, we focus on the influence of illumination fluctuation noise on the image reconstructions. The spatial light field distribution of noisy illumination source acting on the object is equivalent to the object plus the spatial light field distribution of illumination fluctuation noise [40]. Here, we calculate the SNR of the illumination light field, which is defined as ${\mathrm{SNR}}_{I}=10\log_{10}\frac{\langle I(j)\rangle }{\mathrm{Std}(\mathrm{noise})}$, where I(j) denotes the spatial light field of illumination and Std stands for the standard deviation. The properties of MSSIM under different illumination fluctuation noise are presented in Figure 6, from which we can see that under the same ${\mathrm{SNR}}_{I}$, our CC method outperforms DCS and SCS, and all of them can suppress a part of noise with the MSSIM value proportional to the ${\mathrm{SNR}}_{I}$ value.

Next, another simulation was made to see the applicability of our method for the object images at large pixel scale. To rule out that CC only works best for images similar to the head phantom, some different gray-scale original objects were chosen from the open access standard test image gallery. Here, images of a man (Figure 7a), mandrill (Figure 7g) and peppers (Figure 7i) were used, all having the same resolution of 1024×1024 pixels. The reconstructions of the man image were performed at the sampling ratio set from 0.78% to 12.50%, with a 2× stepping increase, as shown in Figure 7b–f. Since these results were retrieved with the same CC method, it is acceptable to still use the RE and PSNR as the quality evaluation criterion. From the results, we can see that the image quality and the calculation time increase with the sampling ratio. Then, the reconstructions (see Figure 7h,j) of different object images with a 12.5% sampling ratio aim to simulate the imaging for general scenes. For color-scale cases, we chose our school badge (Figure 7k) as the object, which was split into the red, green and blue layers. By synthesizing the recovered images of the three wavelength components, the reconstruction of the color image can be obtained, as shown in Figure 7l. The result shows that a multi-wavelength composite image can be reconstructed clearly with 255 tones with little color distortion. The full-color experiments can be easily performed with three spectral filters to CC sample the red, green, and blue sub-images, and then synthesize the three recovered sub-images to form a color image. The simulations in Figure 5 and Figure 7 were all performed with additive white Gaussian noise (its mean is 1% of the measured values mean, and its variance is 1).

Next, we investigated the average performance of our method as a function of image size for a fixed number of different object samples. Here, we picked 12 object samples from the international public standard test image library, and they are airfield, airplane, baboon, barbara, bridge, cablecar, fingerprint, goldhill, man, monarch butterfly, pens, peppers, starfish, and sailboat, respectively, indicated by Obj IDs 1–14. It is known that any natural image has a sparse representation in some basis. As mentioned above, we defined a scalar “sparsity ratio” as the ratio of the number of non-zero (large-value) elements of the image sparse representation coefficients to the total number of image pixels. Since the TVAL3 solver was utilized here, we could use the total variation (TV) operator to sparsely represent the images and calculate the averages of their sparsity ratios at different image sizes. After that, we tested the PSNR performance of our method for different compressive ratios and 12 object samples, compared with RH, DCS, SCS methods. Then, we chose a fixed PSNR value, about 20.334 dB. In this case, we could acquire the lowest required sampling rates of these methods for different image sizes that make the details of the image just be resolved. In Figure 8, we plot the lowest required sampling ratios of above four methods as a function of image size, each point is obtained by averaging 12 results of different object samples. From the graph, we can also see that the images become sparser with higher pixel resolution because they can be approximated even better by low-frequency components, particularly at higher resolutions. Furthermore, the high-frequency component at a low resolution will eventually become a low-frequency one at higher and higher resolutions for the same image. This is a general rule that we can find when looking at compressed versions of megapixel images. That is why we can acquire smaller sampling ratios for different methods at higher resolutions. However, it can be clearly seen from the curves that our CC approach can reduce the sampling rate to a much lower level than other existing advanced methods when obtaining a fixed imaging quality. It is worth mentioning that, although SCS is a very intuitive technology, it needs to fully sample the image or pre-sample the object and then judge whether the measured value should be stored for each measurement (each logic judgment is actually based on the sampling), and the actual number of measurements is still very high. In short, among these four methods, RH performs the worst, while our CC method wins by a landslide, especially for large-scale images, realizing super sub-Nyquist sampling.

It should be noted that, as long as the computer configuration is high enough, the reconstructed images with much larger pixel size (spatial resolution) can also be achieved. Although high-resolution CS reconstruction can be seen in previous work [41,42,43] by using Kronecker product between arbitrary matrices, structurally random matrix, and Hadamard- and circulant-based sensing matrices, our work provides a new way to further reduce the sampling ratio and offers a new insight into the nature of sampling, also with the same benefits of computational efficiency.

### 3.2. Experimental Setup and Results

In our experimental setup, as shown in Figure 9a, the object was illuminated by the collimated and attenuated thermal light beam emitted from a stabilized halogen tungsten lamp, whose wavelength range covers from 360 nm to 2600 nm. Some 2 inch × 2 inch neutral density filters (NDFs) were used to attenuate the light to the ultra-weak light level. The transmission light from the object vertically illuminated a DMD via an imaging lens. The reflected light from the DMD in $-24^{\circ }$ direction with respect to the normal incidence input beam was then sampled by a counter-type Hamamatsu H10682-210 photomultiplier tube (PMT). Since the PMT records the total intensity in the form of photon counts, it can be regarded as a single-photon single-pixel (bucket) detector. Our 0.7 inch DMD (ranging from 350 nm to 2700 nm) consisted of 1024×768 pixels, each of size 13.68 μm×13.68 μm. The states “on” and “off” of the micromirrors were determined by a preloaded sequence of binary patterns. The onboard storage of the DMDs is loadable for up to 45,000 patterns. We independently developed an improved DMD which enables us to load the pattern sequence onto the DMD in real time, releasing restrictions on the onboard memory of the DMD. Here, we used the CC sequence to generate our DMD modulated basis patterns.

The elements of the Hadamard matrix *A* take values of 1 or −1, while the binary patterns encoded on the DMD consist of the values of 1 or 0, which cannot ensure a good image quality with respect to the RIP. It was found that by subtle shifting and stretching operations the matrix *A* can be divided into two complementary matrices $\hat{A}=\left(A+1\right)/2$ and $\overset{ˇ}{A}=1_{N\times N}-\hat{A}$, where 1 stands for an array consisting all ones [44]. Hence, the Hadamard matrix in our optimal ordering can be modulated on the DMD by displaying each complementary basis pattern pair: one pattern shaped from $\hat{A}$ immediately followed by its complementary pattern shaped from $\overset{ˇ}{A}$, i.e., the micromirror states “on” and “off” are reversed. Then, the value range of the differential patterns $A=\hat{A}-\overset{ˇ}{A}$ will become either 1 or −1, actually realizing “positive–negative” intensity modulation as well as differential measurements with a mean ∼0. This method is also named complementary or differential compressed sensing [19].

For simplicity, we tested our system with a gray-scale object. Here, we chose a negative 1951 USAF resolution test chart as the original object (see Figure 9b), whose black parts block the light and white parts transmit the light. The 1951 USAF resolution test chart consists of a series of stripes decreasing in size, while the standard target element is composed of two sets of lines, each set being made up of three lines separated by spaces of equal width. Suppose *r* is the number of lines per mm, the parallel lines are 2.5/r mm long and 0.5/r mm wide with space 0.5/r mm wide between the parallel lines. The space between the vertical and horizontal lines is 1/r mm wide. The elements within a group are numbered from 1 to 6, which are progressively smaller. The group number covers from −2 to 7. The length of any target element line can be expressed as $2.5/2^{\mathrm{Group}+(\mathrm{Element}-1)/6}$mm, while the width equals the length divided by 5, also is equivalent to 0.5/Resolution (line pair/mm). In Figure 9b, the red square is the object image projected on the central square region of our DMD, covering 512×512 pixels (micromirrors). Note that the red square is in Group −1; it is easy to compute the width of each line in the red square (all in Group −1), i.e., 793.70 μm for Element 3, 707.11 μm for Element 4, and 629.96 μm for Element 5.

In our experiments, we first used a CC sequence of 64×64-pixel Hadamard basis patterns, where each pixel unit of the pattern comprises 8×8 adjacent micromirrors. By utilizing our strategy, a coarse image of the object with a low-resolution of 64×64 pixels was retrieved from 1024 patterns, i.e., with a sampling rate of 25%, as shown in Figure 9c. Then, by using a NDF of transmissivity 0.001, Figure 9(d1–d10) illustrates the reconstructed images of 512×512=262,144 pixels by using a series of sampling ratios with a coverage from 0.2% to 100%, where the piece numbers are in their ascending order. From the results, we can see that the lowest sampling ratio in our experiments is about 0.2%, i.e., 524 measurements for 512×512 pixels. For some sparser objects, all the sampling rates can be lower. However, among these approaches, our method provides a shortcut to make the minimum number of measurements, considering the actual sparsity ratio of the object, much better than the cases of random measurements, as shown in Figure 9(f1–f7). It is known that the DMD is the fastest spatial light modulator for the time being, and the nominal maximal binary pattern switching rates of the commercially available DMDs reach 32,550 Hz (patterns/s). With such super sub-Nyquist sampling ratio (0.2% for example), it is available for ∼124 frames per second (fps) at 256×256 pixel resolution, ∼31 fps at 512×512 pixels, and ∼10.35 fps at 1024×768 pixels (regular pixel dimension of the DMD), incorporating two adjacent complementary patterns for differential measurements. Such frame rate is more than enough for real-time applications. In our experiments, we set the modulation frequency to 20 kHz, the acquisition time of the 512×512-pixel image with 0.2% sampling ratio only takes 0.052 s, and the averaged computation time takes about 100 s. If a supercomputer with parallel processing is used or a system hardware is realized, the computation time will be much shorter, in order of milliseconds. In many scenarios, the measurement time is more demanding than the reconstruction time. Therefore, we have reasons to believe that our method can be applied to replace many commercial cameras such as mobile phone cameras in the near future.

Figure 9(e1–e4) shows some comparative examples of using the descending order of piece numbers with a sampling ratio changing from 0.78% to 50%. All their performances are poor, consistent with the simulation results in Figure 2f. The reason is that the patterns with the largest number of connected regions often play the least significant roles. Imagine the connected regions are infinitely subdivided, the most extreme case is that the size of each connected regions is one pixel. Use of such kind of pattern to cover or to modulate the object, the measured value tends to be an average with some negligible fluctuations. However, according to the theory of linear programming, the measured values should have some significant fluctuations around the mean. In contrast, when the patterns tend to be lumped, the corresponding measured values will be larger than the average value with a higher probability, i.e., the correlation between these patterns and the target image is higher. Thereby, the smaller is the number of connected domains of one pattern, the more significant is the contribution of this patterns to the image reconstruction.

Now, let us recall the multiscale/pyramid methods used for image representation, such as wavelet transforms. A Hadamard basis pattern with a smaller number of connected regions (or pieces) actually captures low frequency characteristics of the object image, which is essential for a rough but representative reconstruction, while those patterns with larger piece numbers will undoubtedly add the details of high frequency components by choosing the cutoff sampling percentage if the measurement budget allows. This theory can also explain how our method works.

For performance comparison, we also present the reconstructed images using RH sensing matrix, with the sampling ratios changing from 1.56% to 100.00% with a 2× stepping increase, as shown in Figure 9(f1–f7). From the results, we can see that the image qualities of RH approach with the same sampling ratios are much worse than those of our method. Since neither CS nor DCS can recover any image at such low sampling ratios, SCS requires full sampling, and RDCS cannot generate RD sequence at such high pixel resolution, so their experimental results are not presented here.

Since the SPI has distinct advantages in ultra-weak light noisy measurement environment, we also tested the performance of our CC method in this case, providing the reconstructed results under different SNRs (see Figure 9(g1–g4)) with a same sampling ratio of 3.125%. They were performed by applying four different NDFs, whose transmissivity is 0.001, 0.0025, 0.005 and 0.01 (from left to right), respectively. In these experiments, there was no obstacle placed in the light path to block the light. From the results, we can conclude that our approach is applicable to high (even higher) SNR scenarios. The image quality decreases as the transmissivity of NDFs increases, because the more the light is transmitted, the larger the stray light reflected from the metal surface of the DMD will be.

In view of the fact that the SPI only requires using a single-pixel detector to collect the total light intensity to complete the task of array detection, it has an inherent advantage of being able to resist turbulence. Although the turbulence has a great impact on the spatial light field distribution, it only plays a role of attenuating the total light intensity. To test the performance of our CC approach under turbulence condition, we placed some pieces of lens cleaning tissues (some kind of organic fiber and can be treated as the turbulent obstacle here) between the object and the PMT, as shown in Figure 9a. The number of tissues was increased from one to three and the results are presented in Figure 9(h1–h3), also with a sampling ratio of 3.125%. Some part of the light reflected from the DMD passes through the tissues, and is partly scattered. Thus, the light collected by the PMT is a mixture of the direct light and the indirect light. Despite the detector being completely obscured, our system still retrieved the object through the turbulent obstacles. Thus, the results demonstrate the potential of our system for revealing objects obscured behind obstacles (e.g., foliage) or other scattering materials in actual scenarios.

## 4. Discussion

In our system, the noise cannot be neglected. There are many sources of noise, such as the ambient illumination noise induced by the light source (with temperature drift), the dark noise of the PMT, the stray light reflected from the metal frame of the DMD, the specular and diffuse reflections from the metal surface under the intervals and flipping gaps of micromirrors (the latter is also associated with the patterns), the stray light that bounces back and forth between the metal surfaces, and so forth. Here, the differential measurements can also be used to average the variance of independently and identically distributed noise, thus improving the measurement SNR, as well as the image quality. If other kinds of stochastic noise are well suppressed, the imaging performance will be further improved.

Since the pulse-pair resolution of the used PMT is 20 ns, far shorter than the pattern displaying time of the programmable DMD, the frame rate of image acquisition is actually restricted by the modulation rate of the DMD. It is worth noting here that, for sparser objects, these performance parameters can be much better, and vice versa. Therefore, for relatively low-resolution applications, it allows video rate image acquisition. By making full use of the structured characteristic of Hadamard matrix, we have demonstrated that our method is capable of reconstructing large pixel-resolution images with a high performance, but only needs little computational overhead and memory consumption. Thus, the realization of the system hardware in the future will bring the single-pixel imaging closer to practical applications, including video, night vision goggles and satellites.

The proposed technique employs “cake-cutting” strategy for the Hadamard basis sorting. We certainly believe that the optimized sort sequence of the patterns can be produced by other new methods in the near future. It is worth noting that the orthogonality of Hadamard matrix is the key (but not the only) factor of fast computation and its combination with CS ensures a perfect reconstruction from super sub-Nyquist measurements even in the presence of noise. The design of other orthogonal matrices or deterministic sampling matrices will be our future work. Additionally, the applications in non-visible regions of the spectrum, such as in terahertz, infrared, ultraviolet, and X-ray wavelengths, where the array detectors are not well developed, will be of great interest.

All above data were analyzed and processed with MATLAB R2018b (the MathWorks, Inc.). The ordering and reconstructions were performed on a standard desktop computer with an Inter Core i7-6700 CPU @ 3.40 GHz and a memory of 16 GB.

## 5. Conclusions

We propose a single-pixel compressive imaging method based on “cake-cutting” Hadamard basis ordering, which is capable of precisely reconstructing images of large resolution from super sub-Nyquist measurements. The sampling ratio can be shortened to a much lower level than other existing methods when obtaining the same imaging quality, thus our method significantly reduces the acquisition time. According to the significant contribution of the deterministic Hadamard basis to the image reconstruction, an optimized sequence of patterns is achieved by directly rearranging the internal piece numbers of basis patterns in their ascending order. By taking full advantage of the structured characteristic of Hadamard matrix, the predetermined patterns can be called in real time according to the cake-cutting ordering sequence, without the need to be all stored on the DMD or the computer, thus offering high computational efficiency along with a small computational memory requirement (in computer). Thereby, it tactfully and seamlessly combines super sub-Nyquist sampling with large-image CS reconstruction. Then, we demonstrated this method with a single-photon single-pixel camera based on differential modulation of the DMD. In our experiments, with the maximal pattern switching rates of the commercially available DMDs, the frame rates of the image acquisition could reach ∼31 fps for 512×512 pixels and ∼10.35 fps for 1024 ×768 pixels, which is quite suitable for real-time applications. The experimental results prove that our technique enables a good image reconstruction from CC measurements through a partially obscuring scene in the presence of noise or under ultra-weak illumination. The technique offers an avenue to overcome the limitations existing in the current SPI schemes.

References

## Figures and Tables

**Table 1:** Comparison of the order sequence generation time (s) for RD and our cake-cutting method.

*n*	64	256	1024	4096	16,384	65,536
p=q	8	16	32	64	128	256
**RD**	0.0126	0.0957	2.2301	103.0852	5818.4470	too long
**CC**	0.0052	0.0232	0.1629	2.0058	33.8586	3220.034
${\mathrm{CC}}_{\mathrm{rule}}$	0.000046	0.000051	0.000055	0.000111	0.000224	0.000562
